# Gene Expression Alterations in Peripheral Blood Following Sport-Related Concussion in a Prospective Cohort of Collegiate Athletes: A Concussion Assessment, Research and Education (CARE) Consortium Study

**DOI:** 10.1007/s40279-023-01951-9

**Published:** 2023-11-08

**Authors:** Edward Simpson, Jill L. Reiter, Jie Ren, Zhiqi Zhang, Kelly N. Nudelman, Larry D. Riggen Jr., Michael D. Menser, Jaroslaw Harezlak, Tatiana M. Foroud, Andrew J. Saykin, Alison Brooks, Kenneth L. Cameron, Stefan M. Duma, Gerald McGinty, Steven Rowson, Steven J. Svoboda, Steven P. Broglio, Michael A. McCrea, Paul F. Pasquina, Thomas W. McAllister, Yunlong Liu, CARE Consortium Investigators, Darren Campbell, Jonathan Jackson, Megan Houston, Christopher Giza, Joshua Goldman, Kevin Guskiewicz, Jason P. Mihalik

**Affiliations:** 1 Center for Medical Genomics, Indiana University School of Medicine, Indianapolis, IN USA; 2 Department of Medical and Molecular Genetics, Indiana University School of Medicine, 410 W 10 St, Suite 5000, Indianapolis, IN 46202 USA; 3 Department of Biostatistics and Health Data Science, Indiana University School of Medicine, Indianapolis, IN USA; 4 Department of Epidemiology and Biostatistics, Indiana University School of Public Health, Bloomington, IN USA; 5 Department of Radiology and Imaging Sciences, Indiana University School of Medicine, Indianapolis, IN USA; 6 Department of Orthopedics, University of Wisconsin, Madison, WI USA; 7 Department of Orthopaedic Surgery, Keller Army Community Hospital, United States Military Academy, West Point, NY USA; 8 Department of Physical Medicine and Rehabilitation, Uniformed Services University, Bethesda, MD USA; 9 Institute for Critical Technology and Applied Science, Virginia Tech, Blacksburg, VA USA; 10 United States Air Force Academy, Colorado Springs, CO 80840 USA; 11 Department of Biomedical Engineering and Mechanics, Virginia Tech, Blacksburg, VA USA; 12 Michigan Concussion Center, University of Michigan, Ann Arbor, MI USA; 13 Department of Neurosurgery, Medical College of Wisconsin, Milwaukee, WI USA; 14 Physical Medicine and Rehabilitation Training, Walter Reed Army Medical Center, Washington, DC USA; 15 Department of Psychiatry, Indiana University School of Medicine, Indianapolis, IN USA

## Abstract

**Background:**

Molecular-based approaches to understanding concussion pathophysiology provide complex biological information that can advance concussion research and identify potential diagnostic and/or prognostic biomarkers of injury.

**Objective:**

The aim of this study was to identify gene expression changes in peripheral blood that are initiated following concussion and are relevant to concussion response and recovery.

**Methods:**

We analyzed whole blood transcriptomes in a large cohort of concussed and control collegiate athletes who were participating in the multicenter prospective cohort Concussion Assessment, Research, and Education (CARE) Consortium study. Blood samples were collected from collegiate athletes at preseason (baseline), within 6 h of concussion injury, and at four additional prescribed time points spanning 24 h to 6 months post-injury. RNA sequencing was performed on samples from 230 concussed, 130 contact control, and 102 non-contact control athletes. Differential gene expression and deconvolution analysis were performed at each time point relative to baseline.

**Results:**

Cytokine and immune response signaling pathways were activated immediately after concussion, but at later time points these pathways appeared to be suppressed relative to the contact control group. We also found that the proportion of neutrophils increased and natural killer cells decreased in the blood following concussion.

**Conclusions:**

Transcriptome signatures in the blood reflect the known pathophysiology of concussion and may be useful for defining the immediate biological response and the time course for recovery. In addition, the identified immune response pathways and changes in immune cell type proportions following a concussion may inform future treatment strategies.

**Supplementary Information:**

The online version contains supplementary material available at 10.1007/s40279-023-01951-9.

## Key Points

**Table:** 

This study details the consequences of concussion on changes in gene expression, biological processes, signaling pathways, and immune cell proportions across multiple time points in hundreds of collegiate athletes.
The signaling pathways activated in response to a concussion, along with changes in immune cell proportions inferred through deconvolution analysis, indicate a coordinated immune response that can remain dysregulated long after injury.
These findings are important because the transcriptome signatures of concussion reported herein reflect the known pathophysiology of this injury and reveal the immediate biological response and time course for recovery.

## Introduction

Concussion is a type of mild traumatic brain injury, caused by a blow to the head or a hit to the body, that causes a sudden movement of the head and brain. The sudden movement of the brain inside the skull injures neural cells and blood vessels, resulting in altered brain chemistry and brief loss of normal brain function [1]. Evaluation for a suspected concussion immediately after injury typically involves a clinical examination supported by a symptom assessment and evaluation of motor control and cognitive functioning. Common symptoms include nausea, headache, blurred vision, or confusion, and some individuals may experience a brief loss of consciousness [1]. Symptoms attributed to concussion are most common within the first 7–10 days after injury and for most patients are resolved by 1 month, although a minority of patients may have symptoms that persist for several months or longer [2–4].

Concussion is a common injury in many collegiate sports [5]. Unfortunately, concussions are underreported, not only because the symptoms can be subtle or may not be apparent immediately following injury, but also because some athletes want to remain in competition [6, 7]. Rapid identification of concussion is important because a delay in diagnosis can prolong recovery [8]. Additionally, individuals who return to play before they are fully recovered are at increased risk of sustaining another more serious brain injury [9]. Because of the challenges related to concussion diagnosis, molecular-based approaches that provide complex biological information are necessary to advance concussion research and identify potential diagnostic and/or prognostic biomarkers of injury, as well as measures of recovery.

Given that blood-based assays have identified potential protein biomarkers of concussion injury, we hypothesize that gene expression-based diagnostic or prognostic biomarkers may also exist [10]. Our objective in this study is to identify longitudinal gene expression changes in peripheral blood that are initiated post-injury and are relevant to concussion response and recovery. We analyzed samples from the Concussion Assessment, Research and Education (CARE) Consortium, which was formed to further the study of concussion neurobiology and the consequences of exposure to repetitive head impacts [11]. We anticipate that post-injury gene expression signatures will lead to informative biological processes for long-term recovery prognosis.

RNA sequencing (RNA-seq) is a powerful experimental approach that summarizes the transcriptome of cells and can be used to infer the expression of genes in a tissue or blood sample. This study describes the initial findings from RNA-seq analysis on concussed individuals, spanning preseason baseline and multiple post-injury time points. This study also introduces a comprehensive dataset that will be available publicly and serve as a valuable resource for researchers investigating the consequences of head impacts, traumatic brain injuries, and gene expression biomarkers.

## Methods

### Study Participants and Sample Collection

The full CARE Consortium study description, along with the concussion criteria and recovery protocol, has been detailed earlier [11]. For this study, the CARE Consortium Advanced Research Core provided whole blood samples that were collected from a cohort of 552 civilian varsity and military service academy cadet/midshipmen athletes participating in various collegiate sports between 2015 and 2019. Samples were drawn into PAXgene tubes (BD Biosciences, Franklin Lakes, NJ) at six time points: preseason baseline (Base), at the start of the athletic season before injury; post-injury (PostInj), taken within 6 h of injury; 24–48 h (24-48 h) taken between 24 and 48 h after injury; asymptomatic (Asymp), when an athlete begins return-to-play progression; 7 days post unrestricted return to play (7PostUR); and 6 months (6Mo) from the date of injury [11]. Athletes were divided into three groups based on injury status: injured (INJ) athletes, who sustained a concussion; contact controls (CCT), athletes who participated in contact sports and were teammates of the injured participant but did not sustain a concussion; and non-contact controls (NCC), athletes who did not participate in contact sports [11]. In CCTs, the time span for blood draws at time points after Base was approximated by pairing an individual with an INJ athlete on the same team. Civilian varsity athletes in the INJ group were matched on a 1:1 ratio with teammates who did not incur concussions. Military academy athletes in the INJ group were matched on a 4:1 ratio with teammates who did not incur concussions. Enrollment of the CCT teammates was conducted as close to the injury date as possible. Matching criteria for the INJ and CCT groups were based on institution, sex, sport, Wechsler Test of Adult Reading (WTAR) score, and, for football players, position category. NCC and INJ athletes were matched by institution and sex. We removed any individual from the CCT and INJ groups who did not have a baseline sample. As no preseason baseline blood draws were collected for the NCCs, these individuals were not paired with any other participant for timespan matching. During collection, the time between NCC blood draws was approximated against the distribution of time between INJ follow-up appointments at the discretion of the institution managing the respective NCC participant. During analysis, the initial blood draw for each NCC participant was redefined as Base. Other blood draws for the same NCC participant were then paired separately with the Base sample.

### Sequencing Library Preparation

Total RNA was extracted from blood cells using the PAXgene Blood RNA kit (Qiagen) followed by DNase I treatment to remove contaminating genomic DNA. Dual-indexed strand-specific cDNA libraries were prepared from eluted total RNA using the Kapa mRNA HyperPrep kit (KapaBiosystems) along with QIAseq FastSelect Human Globin removal kit (Qiagen, Germantown, MD). Libraries were prepared in a 96-well plate using a Biomek FxP Laboratory Automation Workstation. Each plate was pooled using the QIAgility Automation System. Pooled libraries were loaded onto a flowcell that was sequenced with 2 × 150 bp paired-end configuration on a NovaSeq 6000 instrument (Illumina, Inc., San Diego, CA).

### Gene Expression Quantification and Differential Expression Analysis

Sequence reads from RNA-seq experiments were aligned to the human genome (hg38) using STAR v2.5.2b. Gene expression levels were quantified by counting the number of RNA fragments aligned to exonic regions of genes using the program featureCounts [12]. The data were analyzed as individual time points compared with baseline to avoid eliminating participants with missing time point data. Differential expression analysis was performed with edgeR using negative binomial generalized log-linear modeling (GLM) and likelihood ratio tests [13, 14]. When calculating distribution parameters with the estimateDisp function, the robust option was used to nullify extreme outliers. Genes with very low read counts were removed before differential expression analysis to reduce the number of individual statistical tests performed and to avoid inflated significance values. Genes were filtered if they had less than 1 count per million mapped fragments (CPM) in a minimum number of samples at each time point. Given the large number of samples in each group, we defined the minimum number of samples as 25% of the smallest group in the comparison (i.e., INJ versus CCT). At each time point, the minimum sample thresholds (*N*) were as follows: PostInj (48), 24-48 h (58), Asymp (61), 7PostUR (57), and 6Mo (42).

Differential expression analysis was performed separately at each post-injury time point adjusting for paired baseline measurements. Let $n$ be the number of subjects and $t=0,\dots ,5$ index the time points from baseline to 6Mo. We denote $E\left({Y}_{it}\right)={\mu }_{it}$, where ${Y}_{it}$ is the gene expression for the $i$th subject ($i$ = 1,…, *n*) at time $t$. To evaluate the differential expression between groups at the $j$th post-injury time point ($j$ = 1,…, 5), the model can be expressed as

$$\mathrm{log}\left({\mu }_{it}\right)=\mathrm{log}\left({M}_{it}\right)+{\beta }_{i}+{{\varvec{\gamma}}}^{\prime}{{\varvec{x}}}_{i}I(t=j),$$

$t=0, j$, where $I(\cdot)$ is an indicator function, ${M}_{it}$ is the effective library size, ${\beta }_{i}$ is the individual intercept, and ${{\varvec{x}}}_{i}={({x}_{i1},{x}_{i2},{x}_{i3})}^{\prime}$ is a vector of group indicators. ${x}_{i1}=1$ if the subject $i$ was in the INJ group and ${x}_{i1}=0$ otherwise. Similarly, ${x}_{i2}$ and ${x}_{i3}$ are the indicators for CCT and NCC groups, respectively. The coefficient vector ${\varvec{\gamma}}={({\gamma }_{1},{\gamma }_{2},{\gamma }_{3})}{^\prime}$ represents the corresponding group effects at the $j$ th post-injury time point. The difference in changes from the baseline across the CCT and INJ groups can be evaluated by contrasting ${\gamma }_{1}$ and ${\gamma }_{2}$. Background levels of gene expression differences at the PostInj time point were defined by comparing CCT and NCC participants. The within-subject variation over time observed in NCC participants was assumed to arise from natural fluctuations in gene expression. Therefore, all time points following the initial blood draw for the NCC group were treated as the PostInj time point. In all comparisons, genes with Benjamini–Hochberg false discovery rate (FDR) ≤ 0.05 were considered significant.

### Gene Ontology Analysis

Gene Ontology (GO) analysis is a type of enrichment analysis where the top differentially expressed genes in an experiment, defined by a significance cutoff, are matched against reference gene lists that have been annotated to biological terms or functions. Enrichment tests determine if a term is significant by comparing the number of matched genes in a list to a random background. GO analysis was performed in R using the clusterProfiler package [15]. Differentially expressed genes with FDR ≤ 0.05 were converted to Entrez gene IDs using Biomart [16]. Term enrichment at the PostInj time point was determined using clusterProfiler to search the following reference lists: biological process, cellular component, molecular function, and Kyoto Encyclopedia of Genes and Genomes (KEGG). Only terms with Benjamini–Hochberg FDR ≤ 0.05 were considered significant. Terms with enrichment lists containing ≥ 50% common genes were merged.

### Gene Set Enrichment Analysis

Gene set enrichment analysis (GSEA), which is not dependent on a significance cutoff, is used when few or no statistically significant differentially expressed genes have been identified. Instead, genes are ordered by significance and a running score is obtained as matching proceeds down the full list. Differentially expressed genes at all time points were ranked by the − log(*p*-value) from differential expression analysis multiplied by the sign of the fold change and analyzed using the GSEA v4.1.0 app and MSigDB v7.3 [17–19].

### Deconvolution Analysis

Deconvolution analysis is the process where cell type proportions can be estimated from bulk RNA-seq data based on marker gene expression. Deconvolution analysis was performed with CIBERSORTx using raw read counts and default software normalization [20]. A GLM was used to test the difference between estimated percentages of cell types output by CIBERSORTx. The cell type percentage of the Base sample and the group were used as covariates to predict the cell type percentage of the time point sample. All time points were tested, and cell types with Benjamini–Hochberg FDR ≤ 0.05 at a given time point were considered significant.

## Results

### Demographics

A total of 2489 blood samples were collected from 552 athletes among all groups. Participants without baseline samples in the CCT and INJ groups were filtered, leaving 130 CCT, 230 INJ, and 102 NCC individuals with a combined total of 2125 blood samples. Demographic data of the participants enrolled in the present study are provided in Table 1. All collegiate athletes participated in a varsity sport, whereas the primary sport for military cadets/midshipmen also included club and intramural sports. Group comparisons of sex, age at baseline, race, and ethnicity were evaluated using Fisher’s exact test for categorical variables and ANOVA for continuous variables. The NCC group exhibited a slightly higher average age (*p* = 0.0441), which can be explained by a higher proportion of athletes participating in contact sports being enrolled at 18 years of age compared with athletes participating in non-contact sports, where the highest proportion was enrolled at 19 years of age.

**Table 1:** Cohort demographics of enrolled CARE participants

Factor	NCC	CCT	INJ	*p*-value
Total	102	130	230	
Sex				0.7004
Male	82 (80.4%)	99 (76.2%)	182 (79.1%)	
Female	20 (19.6%)	31 (23.8%)	48 (20.9%)	
Age (SD)	19.4 (1.3)	19.1 (1.3)	19.0 (1.3)	0.0441
Race				0.2813
African American	13 (12.7%)	30 (23.1%)	45 (19.6%)	
Other	11 (10.8%)	11 (8.5%)	28 (12.2%)	
White	78 (76.5%)	89 (68.5%)	157 (68.3%)	
Ethnicity				0.8870
Hispanic	8 (7.9%)	9 (7.6%)	13 (6.5%)	
Non-Hispanic	93 (92.1%)	109 (92.4%)	188 (93.5%)	
Primary sport				
Football		62 (47.7%)	101 (43.9%)	
Ice hockey		10 (7.7%)	20 (8.7%)	
Soccer		29 (22.3%)	47 (20.4%)	
Lacrosse		8 (6.2%)	16 (7.0%)	
Rugby		9 (6.9%)	27 (11.7%)	
Wrestling		2 (1.5%)	7 (3.0%)	
Cross country/track	37 (36.3%)	1 (0.8%)	2 (0.9%)	
Intramurals		6 (4.6%)	9 (3.9%)	
Softball	7 (6.9%)		1 (0.4%)	
Baseball	37 (36.3%)			
Basketball	13 (12.7%)			
Field event	8 (7.8%)			
Other		3 (2.3%)		
Injury sustained				
Competition			88 (38.3%)	
Practice/training			132 (57.4%)	
Outside primary sport			10 (4.3%)	

*NCC* non-contact control, *CCT* contact control, *INJ* injured athletes who sustained a concussion. “Other” in “Sport” includes skiing, boxing, and handball. “Outside primary sport” indicates concussions that occurred by similar mechanisms as the sport-related concussions and included injuries sustained from physical education class, accidental falls, and accidental kick to the head. *SD* standard deviation

### Sample Set Description

Because some sample collections were missed, unaccounted for, improperly recorded, or failed quality control, the number of samples at each time point differed between groups. During the study, nine CCT participants sustained a concussion and were subsequently reclassified as INJ participants; as a result, samples from these athletes were present in both CCT and INJ groups. Therefore, the baseline samples for these nine participants were duplicated for the reclassified sample sets, while the other blood draws for these participants (40 CCT and 33 INJ) remained unique in the dataset. In addition, one CCT athlete served as a control in two different seasons; the first CCT sample set consisted of five blood draws and the second set consisted of two blood draws. The same baseline sample was used for both of these sample sets.

**Fig. 1:**
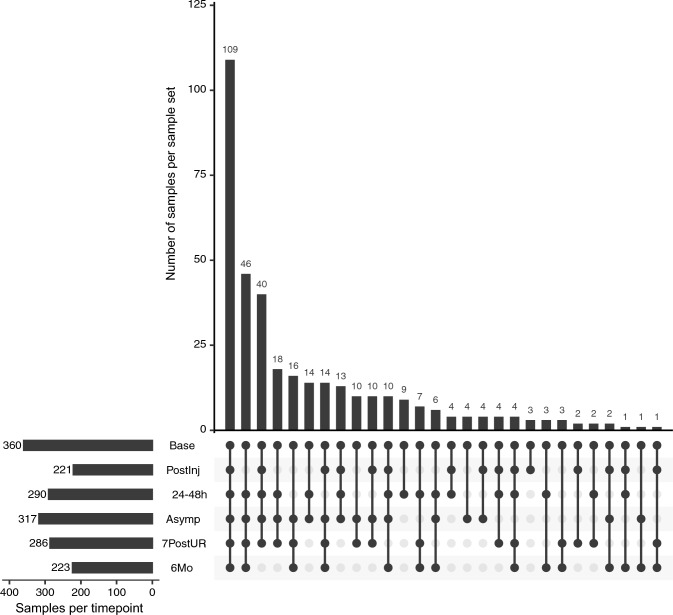
Distribution of sample sets at each time point for contact control (CCT) and injured (INJ) blood samples. The main panel shows the total number of samples with multiple time points. The time point coverage is annotated below. A black dot indicates a blood sample was drawn at a given time point. The left bar plot indicates the total number of samples at each time point. *Base* baseline, *Postinj* post-injury, within 6 h, *24–48 h* taken between 24 and 48 h after injury, *Asymp* asymptomatic, when an athlete begins return-to-play progression, *7PostUR* 7 days post unrestricted return to play, and *6Mo* 6 months from the date of injury

We constructed 130 CCT and 230 INJ sample sets, each having a baseline blood draw and at least one or more samples from a later time point. A summary of sample numbers is provided in Table 2, and the distribution of participant sample sets is shown in Fig. 1. The median (interquartile range, IQR) time of sample collection for the 24-48 h time point was 43.9 h (36.7, 49.0). The median (IQR) time of sample collection for the Asymp and the 7PostUR time points was 6.6 (4.0, 9.9) and 20.2 (16.6, 27.0) days, respectively.

**Table 2:** Sample number for contact control (CCT) and injured (INJ) athletes at each time point

	CCT	INJ
Baseline	130	230
Post injury	125	96
24–48 h	117	173
Asymptomatic	123	194
7PostUR	115	171
6 months	85	138
Total	695	1002

*7PostUR* 7 days post unrestricted return to play

NCC samples were used to represent time-based gene expression variance. For each of the 102 NCC participants, the first sample drawn was designated as the Base sample and each subsequent sample for that participant was individually paired with the Base sample as a separate sample set. As a result, there were 326 NCC sample sets, each with only two samples where the baseline sample may have been duplicated in another NCC sample set from the same athlete.

### Differentially Expressed Genes

**Fig. 2:**
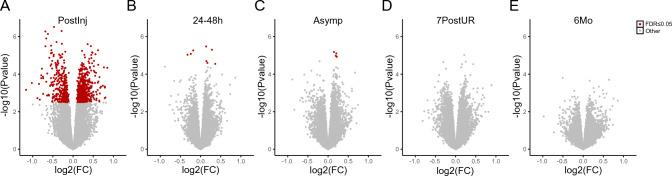
Differentially expressed genes between INJ and CCT groups at each post-injury time point. Volcano plots showing the log_2_ fold change (FC) of differentially expressed genes at each individual time point following concussion injury after adjusting for baseline measurements. Genes with false discovery rate (FDR) ≤ 0.05 are in red. **A** PostInj, post-injury, within 6 h; **B** *24–48 h* taken between 24 and 48 h after injury; **C** *Asymp* asymptomatic, when an athlete begins return-to-play progression; **D** *7PostUR* 7 days post unrestricted return to play; **E** *6Mo* 6 months from the date of injury

To investigate how sport-related concussion altered gene expression patterns in peripheral blood over time, we performed differential gene expression analysis on the RNA-seq data at each of the sampled time points. The highest number of differentially expressed genes occurred at the PostInj time point (*N* = 860, FDR ≤ 0.05) and that number was reduced 100-fold by the 24-48 h time point (*N* = 8). Volcano plots of differentially expressed genes at all follow-up time points are shown in Fig. 2. Lists of differentially expressed genes, fold changes, and significance values for each time point are provided in Online Resource 1.

A known consequence of brain injury is damage of neuronal cell membranes that triggers ionic flux and disrupts calcium metabolism and calcium-dependent signaling [21]. The cellular response to restore homeostasis entails activating ion pumps, including calcium pumps, which in turn consume ATP and starve the brain of energy [21]. Among the differentially expressed genes, we observed that multiple genes related to calcium metabolism were altered at the PostInj time point, including *CAMK2G*, *CAMKK2*, and *CAMKK1*, which were all upregulated in INJ participants. Additionally, expression of many solute transporters was altered, including four members of the SLC22 family (*SLC22A15*, *SLC22A16*, *SLC22A1*, and *SLC22A4*) that transport carnitine, which is used in cells to transport long-chain fatty acids into mitochondria for energy production [22]. Together, the upregulated genes we observed related to calcium and energy metabolism suggested a compensatory effect following injury, and matched the pathophysiology reported for concussions.

We also investigated gene expression of known potential protein biomarkers for traumatic brain injury diagnosis [23]. Genes for two FDA-approved traumatic brain injury biomarkers used in the i-STAT TBI plasma test (Abbott), *GFAP* and *UCH-L1*, did not meet the minimum expression threshold for analysis at any time point. The *MAPT* gene encoding Tau also did not meet the minimum expression threshold for analysis at any time point. *NEFL*, encoding neurofilament light chain, was expressed at all time points, but no significant differences were observed between INJ and CCT participants.

### Pathway Enrichment Following Concussion

**Fig. 3:**
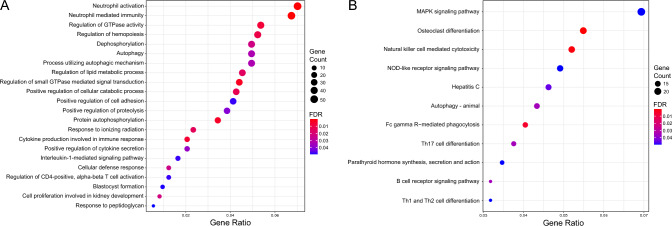
Biological pathway enrichment following concussion. Significantly enriched Gene Ontology (GO) (**A**) and Kyoto Encyclopedia of Genes and Genomes (KEGG) (**B**) terms at the PostInjury time point. The diameter of the point indicates the number of differentially expressed genes (FDR ≤ 0.05) matched to a term. Color is scaled from blue to red by increasing significance. Gene ratio refers to the number of genes matched to a term compared with the total number of differentially expressed genes at the PostInjury time point

To explore the biological function of differentially expressed genes after concussion, we performed GO term and KEGG pathway enrichment analysis on the differentially expressed genes at the PostInj time point. The top two biological processes were neutrophil activation and neutrophil-mediated immunity (Fig. 3A, Online Resource 2). Several other significant biological processes were also related to immune response, which is consistent with inflammation as a mechanism of neuronal tissue damage in concussion injuries [24, 25]. In addition, we also observed significant gene expression differences in multiple interleukin receptor genes at the PostInj time point, including *IL1R1*, *IL1R2*, *IL1RAP*, and *IL2RB*, which is consistent with an acute inflammatory response and upregulated cytokine production that has previously been reported in traumatic brain injury studies [26, 27]. Several other biological processes related to signal transduction pathways were found, such as regulation of GTPase activity and protein phosphorylation. Likewise, enriched KEGG pathways included natural killer cell mediated cytotoxicity, MAPK signaling pathway, and NOD-like receptor signaling activity (Fig. 3B, Online Resource 2).

**Fig. 4:**
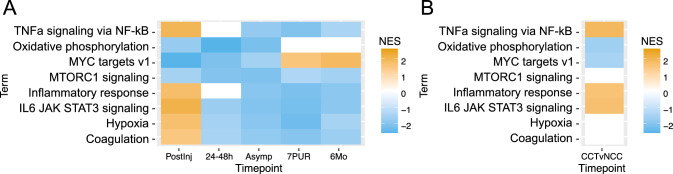
Gene Set Enrichment Analysis (GSEA) using Hallmark gene sets. **A** Normalized enrichment scores (NES) for Hallmark gene sets from GSEA across all time points. **B** NES for Hallmark gene sets using differential expression results from the comparison of the CCT group versus the NCC group at the PostInjury time point. Plotted terms in both figures were filtered from all results by selecting terms that were significant with FDR ≤ 0.05 at the PostInjury time point and also significant with FDR ≤ 1E–5 in at least one other time point. *Postinj* post-injury, within 6 h, *24–48 h* taken between 24 and 48 h after injury, *Asymp* asymptomatic, when an athlete begins return-to-play progression, *7PUR* 7 days post unrestricted return to play; and *6Mo* 6 months from the date of injury

The small number of differentially expressed genes after the 24-48 h time point prohibited GO analysis. Therefore, to compare enriched cellular processes and pathways at all time points relative to the baseline, we performed GSEA. Enrichment results using hallmark gene sets are shown in Fig. 4A. Similar to the findings from GO analysis and KEGG pathways, GSEA also showed that the top-ranked pathways immediately following concussion were related to upregulation of immune-related signaling. For example, “*TNFa* signaling via *NF-kB*,” “inflammatory response,” and “*IL6 JAK STAT3* signaling” were all significantly positively enriched (FDR ≤ 0.05), each of which has also been strongly associated with response to concussion [27–32]. In addition, differentially expressed genes identified at the PostInj time point included multiple genes downstream of JAK, such as members of PI3K-AKT, MAPK, and STAT signaling pathways. These genes include *JAKMIP1*, *JAKMIP2*, *PRR5L*, *MAPK13*, *STAT6*, and *BCL6*. Additionally, two regulatory subunits of protein phosphatase PP2, *PPP2RB2* and *PPP2R5E*, were differentially expressed; PP2, a serine/threonine phosphatase, targets Raf, MEK, and AKT signaling cascade pathways. At later time points, we also observed significant enrichment for “*TNFa* signaling via *NF-kB*,” “inflammatory response,” and “*IL6 JAK STAT3* signaling”; however, the enrichment scores were now negative.

To determine whether the observed reversal in enriched pathway scores at later time points might be related to recovery of injured athletes after being removed from play, we compared enriched processes at the PostInj time point between CCT and NCC participants (Fig. 4B). Interestingly, we observed that the immune signaling processes “*TNFa* signaling via *NF-kB*,” “inflammatory response,” and “*IL6 JAK STAT3* signaling,” were also positively enriched in CCT participants. Therefore, it appeared that athletes participating in contact sports exhibited higher activation of certain immune signaling processes compared with athletes participating in non-contact sports. These immune signaling processes became further elevated immediately following concussion and were then downregulated below CCT levels during recovery. GSEA results also indicated that some altered immune signaling pathways in the INJ group appeared to remain repressed compared with the CCT group for at least 6 months following a concussion (Fig. 4A).

**Fig. 5:**
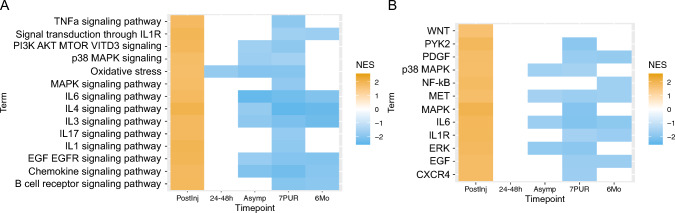
Gene set enrichment analysis using WikiPathways and Biocarta. Normalized enrichment scores (NES) for WikiPathways (**A**) and Biocarta (**B**) gene sets from GSEA across all time points. Plotted terms were filtered from all results by selecting terms that were significant with FDR ≤ 0.05 at the PostInj time point and related to pathways found in GO analysis, Hallmark GSEA, or were otherwise immune-associated in literature searches. *Postinj* post-injury, within 6 h, *24–48 h* taken between 24 and 48 h after injury, *Asymp* asymptomatic, when an athlete begins return-to-play progression, *7PUR* 7 days post unrestricted return to play, and *6Mo* 6 months from the date of injury

To confirm our observations from the GSEA Hallmark gene lists, we also performed GSEA with gene lists from WikiPathways and Biocarta [33, 34]. Similar findings were observed in both WikiPathways (Fig. 5A) and Biocarta (Fig. 5B), in that gene sets were positively enriched (FDR ≤ 0.05) at the PostInj time point and negatively enriched at later time points. Observed pathways included those associated with cytokine production and inflammatory response. Full results from GSEA analyses are provided in Online Resource 3.

### Altered Immune Cell-Type Proportions Following Concussion

Because we observed differential expression of genes that are important in immune signaling, we asked whether there might be any changes in circulating cell type populations in response to concussion. To address this question, we performed deconvolution analysis on RNA-seq counts using CIBERSORT to identify immune cell proportions. We then evaluated differences in cell type proportions between INJ and CCT groups at all time points using a GLM. Only two cell types at the PostInj time point were determined to be differentially proportioned; neutrophils were more prevalent (FDR 0.023) and natural killer cells were less prevalent (FDR 2.49 × 10^−5^) in the INJ group (Online Resource 4). No differences in cell-type proportions were observed at later time points. Our finding that the neutrophil proportion increased within 6 h following concussion is consistent with a previous report that also found an increase in neutrophils following mild traumatic brain injury at the site of injury [35].

## Discussion

The results of this study provide new insights into the biological responses following concussion injury by comparing gene-expression changes from five post-injury time points against a participant’s baseline sample. We found a high number of gene expression changes in peripheral blood cells immediately following sport-related concussion, many of which were consistent with a major immune signaling response. We also identified compensatory changes in genes associated with calcium and energy metabolism that are consistent with the pathophysiology of concussion. Our finding that expression of genes that mediate immune signal transduction was enhanced after injury was further supported by GO analysis and GSEA. Immune signaling processes initiated immediately following concussion were largely suppressed within 24 h and remained repressed compared with contact controls during the 6-month recovery period. Lastly, we found the proportion of neutrophils in peripheral blood was higher in injured participants compared with contact control athletes.

One strength of our study is that RNA-seq technology allowed quantification of significantly more genes compared with earlier studies that used microarray-based chips. However, some of these earlier small studies noted differentially expressed genes persisted days or months after injury, whereas we found only a few differentially expressed genes 24 h following concussion [32, 36]. One potential explanation for the observed differences in gene expression at later time points is the higher biological variability in our study, which derives from the complex study design that spans multiple sports and institutions. Nevertheless, while a relatively small number of gene expression differences may persist that we could not observe, our findings provide evidence that a short-term surge in gene expression triggers a powerful immune response. This conclusion is consistent with the findings of at least one other study of sport-related concussions [32].

Notably, we did not observe changes in the expression of genes coding for known blood biomarkers of concussion injury. This finding suggests that protein and molecular biomarkers of concussion in peripheral blood are not related to gene expression changes in blood cells, and instead likely result from release by injured tissues, changes in protein metabolism, or other sources. Therefore, RNA-seq of peripheral blood samples may yield complementary yet distinct diagnostic targets compared with blood-based protein biomarker testing.

One limitation of our study is that peripheral blood samples do not reflect the physiological environment of a concussion as well as brain tissue from the site of injury. However, blood is easily accessible for diagnostic testing and therefore is of interest for use in identifying potential biomarkers for concussion diagnosis and monitoring recovery. Another limitation is that individuals who have suffered a sport-related concussion may have also sustained a blow to the body that might have contributed to gene expression changes in blood. We expect, however, that the wide variety of contact sports and the use of teammates as contact controls in this study minimized identification of non-concussion-related gene expression changes. Additionally, while sex is known to influence concussion recovery, we did not investigate the influence of sex on gene expression following a concussion. As our study objective was to identify common gene expression changes, we first compared differential gene expression in individuals with their own preseason baseline sample; thus, the changes we identified are independent of sex. Finally, our study would be strengthened by additional samples and more complete time courses to fully explore the longitudinal effects of concussion on gene expression. Increasing the sample size would increase the statistical power for identifying genes with smaller changes in differential expression and could improve the findings at individual time points as well.

## Conclusion

To our knowledge, this study is the largest concussion transcriptome study to date. Our findings confirm results from several smaller studies and expand on the existing knowledge base by showing trends in gene expression following injury and concussion-related pathways during recovery. We anticipate the dataset we provide herein will be a rich source of information that advances research in new strategies for the treatment of concussion injury.

## Supplementary Information

Below is the link to the electronic supplementary material.

Supplementary file1 (XLSX 3056 KB)

Supplementary file2 (XLSX 44 KB)

Supplementary file3 (XLSX 1703 KB)

Supplementary file4 (XLSX 392 KB)

## Declarations

### Data Availability

Patient metadata and gene expression data in the form of raw counts are accessible as part of CARE 1.0 on the Federal Interagency Traumatic Brain Injury Research Informatics System (FITBIR, fitbir.nih.gov). Raw sequence data are available on dbGap (accession number is pending). Criteria for access are determined by each of the entities hosting the data and the governance of their respective regulatory committees.

### Code Availability

Computer code is available from the authors upon request.
